# HIV diagnoses in migrant populations in Australia—A changing epidemiology

**DOI:** 10.1371/journal.pone.0212268

**Published:** 2019-02-14

**Authors:** Praveena Gunaratnam, Anita Elizabeth Heywood, Skye McGregor, Muhammad Shahid Jamil, Hamish McManus, Limin Mao, Roanna Lobo, Graham Brown, Margaret Hellard, Tafireyi Marukutira, Neil Arvin Bretaña, Carolyn Lang, Nicholas Medland, Benjamin Bavinton, Andrew Grulich, Rebecca Guy

**Affiliations:** 1 Kirby Institute for Infection and Immunity, UNSW Sydney, Sydney, Australia; 2 School of Public Health and Community Medicine, UNSW Sydney, Sydney, Australia; 3 Centre for Social Research in Health, UNSW Sydney, Sydney, Australia; 4 School of Public Health, Curtin University, Perth, Australia; 5 Australian Research Centre in Sex, Health and Society, La Trobe University, Melbourne, Australia; 6 Burnet Institute, Melbourne, Australia; 7 Communicable Diseases Branch, Queensland Department of Health, Brisbane, Australia; 8 Melbourne Sexual Health Centre, Alfred Health, Melbourne, Australia; 9 Central Clinical School, Monash University, Melbourne, Australia; Centers for Disease Control and Prevention, UNITED STATES

## Abstract

**Introduction:**

We conducted a detailed analysis of trends in new HIV diagnoses in Australia by country of birth, to understand any changes in epidemiology, relationship to migration patterns and implications for public health programs.

**Methods:**

Poisson regression analyses were performed, comparing the age-standardised HIV diagnosis rates per 100,000 estimated resident population between 2006–2010 and 2011–2015 by region of birth, with stratification by exposure (male-to-male sex, heterosexual sex–males and females). Correlation between the number of permanent and long-term arrivals was also explored using linear regression models.

**Results:**

Between 2006 and 2015, there were 6,741 new HIV diagnoses attributed to male-to-male sex and 2,093 attributed to heterosexual sex, with the proportion of diagnoses attributed to male-to-male sex who were Australian-born decreasing from 72.5% to 66.5%. Compared with 2006–2010, the average annual HIV diagnosis rate per 100,000 in 2011–15 attributed to male-to-male sex was significantly higher in men born in South-East Asia (summary rate ratio (SRR) = 1.37, p = 0.001), North-East Asia (SRR = 2.18, p<0.001) and the Americas (SRR = 1.37, p = 0.025), but significantly lower as a result of heterosexual sex in men born in South-East Asia (SRR = 0.49, p = 0.002), Southern and Central Asia (SRR = 0.50, p = 0.014) and Sub-Saharan Africa (SRR = 0.39, p<0.001) and women born in South-East Asia (SRR = 0.61, p = 0.002) and Sub-Saharan Africa (SRR = 0.61, p<0.001). Positive associations were observed between the number of permanent and long-term arrivals and HIV diagnoses particularly in relation to diagnoses associated with male-to-male sex in men from North Africa and the Middle East, North Asia, Southern and Central Asia and the Americas.

**Conclusion:**

The epidemiology of HIV in Australia is changing, with an increase in HIV diagnosis rates attributed to male-to-male sex amongst men born in Asia and the Americas. Tailored strategies must be developed to increase access to, and uptake of, prevention, testing and treatment in this group.

## Introduction

Migrants in high income, low prevalence countries can be disproportionately affected by HIV, with higher diagnosis rates and a greater likelihood of being diagnosed late, with a CD4 count under 350 cells/ul [1–3]. Historically migrants from high prevalence countries such as in Sub-Saharan Africa have been the most affected, but emerging evidence from Europe suggests this is changing, with decreases in HIV diagnoses attributed to heterosexual exposure in African migrants and increases in new HIV diagnoses attributed to male-to-male sex in Latin American migrants [1, 2].

Australia has a concentrated HIV epidemic, with around three quarters of new diagnoses each year attributed to male-to-male sex and one-fifth to heterosexual exposure [4]. The number of HIV diagnoses in Australia attributed to sex between men increased from 655 in 2007 to 755 in 2012 and has remained stable since [4]. This stabilisation coincided with promotion of “treatment as prevention” and increases in testing frequency, treatment coverage and viral suppression [4, 5]. However a shift in HIV risk may be taking place, with the proportion of HIV diagnoses attributed to male-to-male sex in overseas-born men who are Asian-born increasing from 30% in 2006 to 57% in 2015 [4]. Simultaneously there have been an increase particularly in younger migrants from Asian countries such as China [6]. India, the Philippines, Viet Nam and Malaysia were also among the top ten countries of birth in Australia in 2016 [6].

Migrants with HIV in Australia have differing histories and reasons for migration. Understanding this diversity is important in designing programs which maximise access to HIV testing, treatment and care to reduce HIV transmission. Australia has committed to working towards elimination of transmission of HIV by 2020 [5], and addressing the needs of overseas-born populations who may be at risk of HIV an important component of achieving this goal.

In this study, we sought to determine if, similar to other high-income countries, the epidemiology of HIV by country of birth and exposure in Australia has changed over time, and if any changes are related to migration patterns. Findings are intended to inform HIV programs for people born overseas.

## Materials and methods

### HIV surveillance procedures

Under procedures described previously [7], all new HIV diagnoses in Australia are first notified by laboratories and/or doctors to State and Territory health authorities, who analyse and report this data to the National HIV Registry, maintained by the Kirby Institute at UNSW Sydney on behalf of the Australian Government Department of Health [8]. Data was de-identified before provision to the lead author for this analysis, with name code (full name is converted to 2x2 code before reporting by states and territories to the national registry) removed and date of birth converted to age at diagnosis. Consent was not sought from individuals as this would have involved re-identification of data and individuals in order to contact them, which in itself could constitute a breach of privacy.

Data extracted from the National HIV Registry for this analysis were year and age at diagnosis, sex, country of birth (routinely collected from 2002), mode of exposure to HIV (males who have sex with males (MSM); MSM with dual risk of injecting drug use (IDU); heterosexual sex; and other/undetermined exposure, including mother-to-child transmission, direct blood/tissue exposure, and iatrogenic exposure, likely place of acquisition (Australia or overseas) and year of arrival in Australia.

Countries of birth were grouped into one of eight regions (Australia, Oceania, North-West Europe, Southern and Eastern Europe, North Africa and the Middle East, South-East Asia, North-East Asia, Southern and Central Asia, Americas and Sub-Saharan Africa) based on categories used by the Australian Bureau of Statistics (ABS) [9]. The ABS has primary responsibility in Australia for collection of population data.

The likely place of acquisition and year of arrival variables have only been available nationally since 2015, so analysis of these variables was undertaken using data from this year alone.

Data on estimated resident population (ERP) [10] and permanent and long-term arrivals by year, age, sex and country of birth for each year [11, 12] were sourced from the ABS. Projections of ERP by country of birth are calculated on an annual basis using multiple components, including census count, births, deaths and net overseas migration [13]. Permanent arrivals include travellers who hold a permanent visa (regardless of duration of stay); long-term arrivals include both residents returning from an absence, or visitors with duration of stay of one year or longer [14].

### Analysis

Desk-based, secondary data analysis was conducted. All people newly diagnosed with HIV in Australia between 1 January 2006 and 31 December 2015 with the HIV exposure recorded as male-to-male or heterosexual sex were included. Excluded cases had a prior diagnosis overseas, no country of birth was recorded, and/or were transgender, the last exclusion to allow stratification of risk by sex.

The study period was divided into two equal five-year intervals for comparison of changes over time–(i) 2006 to 2010 and (ii) 2011 to 2015. Descriptive analysis was conducted to calculate the proportions of HIV diagnoses by region of birth, by sex and mode of exposure (male-to-male sex or heterosexual). The Wilcoxon rank sum test was used to compare median age at diagnosis by region of birth and exposure, using people born in Australia as the comparison. Pearson’s chi-squared test was used to compare overseas-born diagnoses by likely place of acquisition and reported exposure.

Average annual HIV diagnosis rates per 100,000 population were calculated by region of birth for each time period, with stratification by exposure (male-to-male sex, heterosexual males and heterosexual females), and age standardisation using the 2001 ABS standard population [15] as a reference. Average annual age-standardised HIV diagnosis rates by region of birth were compared using univariate Poisson models, with the age-standardised number of diagnoses as the outcome, region of birth as the independent variable and ERP as the population offset. Results are presented as an incidence rate ratio (IRR) for each region of birth compared with Australia.

The difference in the average annual age-standardised HIV diagnosis rate between 2006–2010 and 2011–2015 for each region of birth was analysed using univariate Poisson models, with number of diagnoses as the outcome and time interval as the independent variable. Summary rate ratios (SRRs) for each region of birth and exposure category were calculated, using the 2006 to 2010 time period as the reference.

To understand the relationship between migration patterns and new HIV diagnoses, we used linear regression models for each region of birth and mode of exposure, with number of HIV diagnoses per year as the dependent variable and permanent and long-term arrivals as the independent variable. Models were adjusted by including age as a covariate in the multivariate analysis. This analysis provides a more sensitive measure of any relationship between migration patterns and HIV diagnoses than using ERP alone, as ERP is based on population projections whereas permanent and long-term arrivals are real-time data.

For all analyses, the population denominator used for calculation of HIV diagnosis rates in men—whether attributed to male-to-male sex or heterosexual sex—was the overall male population, as population numbers stratified by mode of exposure are not available. This method is consistent with those used in producing national surveillance estimates [4].

All analyses were performed using STATA IC 14 [Stata Corp, College Station, TX], with the significance level set at p>0.05.

### Ethics

Ethical approval was received from the University of New South Wales Human Research Ethics Committee (Reference HC16482).

## Results

### Epidemiological characteristics by region of birth

Table 1 summarises the characteristics of all new HIV diagnoses by region of birth. Between 2006 and 2015, there were a total of 11,451 HIV diagnoses in Australia, of these 9,869 (86.1%) were new diagnoses, with no prior diagnosis overseas. Of these new diagnoses, 9,566 (97%) had country of birth recorded, and 8,840 of 9,566 (92%) reported male-to-male or heterosexual sex as their exposure to HIV. After excluding six transgender individuals, a final sample of 8,834 persons diagnosed with HIV in Australia between 2006 and 2015 were included in the analysis.

**Table 1:** Characteristics of new HIV diagnoses in Australia, 2006–2015.

	Australia		Oceania		North West Europe		Southern and Eastern Europe		North Africa and Middle East		South-East Asia		North East Asia		Southern and Central Asia		Americas		Sub-Saharan Africa		
	N	%	N	%	N	%	N	%	N	%	N	%	N	%	N	%	N	%	N	%	
**2006–2010**																					
Sex																					
Male	2473	67.2	145	3.9	259	7.0	67	1.8	47	1.3	271	7.4	70	1.9	54	1.5	109	3.0	184	5.0	**3679**
Female	120	25.0	33	6.9	16	3.3	6	1.3	19	4.0	111	23.1	7	1.5	6	1.3	8	1.7	155	32.2	**481**
Age (Median)	37 (IQR 29–45)		36 (IQR 27–44, p = 0.037)		41 (IQR 31–52, p<0.001)		43 (IQR 32–55, p<0.001)		34 (IQR 27–40, p = 0.011)		32 (IQR 27–39, p<0.001)		30 (IQR 24–36, p<0.001)		31 (IQR 25–39, p<0.001)		35 (IQR 30–42, p = 0.136)		34 (IQR 29–41, p<0.001)		
Male to male sex	37 (IQR 29–44)		37 (IQR 29–44, p = 0.817)		40 (IQR 31–48, p = 0.001)		37 (IQR 31–51, p = 0.100		35 (IQR 23–41, p = 0.114)		33 (IQR 27–39, p<0.001)		29 (IQR 24–37, p<0.001)		33 (IQR 26–43, p = 0.157)		36 (IQR 30–42, p = 0.241)		35 (IQR 28–45, p = 0.799)		
Heterosexual sex	41 (IQR 31–50)		31 (IQR 25–43), p<0.001)		48 (IQR 38–58, p<0.001)		53 (IQR 43–58, p = 0.003)		33 (IQR 27–40, p = 0.003)		32 (IQR 28–40, p<0.001)		34 (IQR 28–36, p = 0.111)		31 IQR 25–39, p<0.001)		33 (IQR 29–48, p = 0.299)		34 (IQR 29–40, p<0.001)		
Exposure																					
Male to male sex	2227	72.5	127	4.1	206	6.7	52	1.7	22	0.7	210	6.8	68	2.2	20	0.7	98	3.2	44	1.4	**3074**
Heterosexual sex	366	33.7	51	4.7	69	6.4	21	1.9	44	4.1	172	15.8	9	0.8	40	3.7	19	1.8	295	27.2	**1086**
**Total**	2593	62.3	178	4.3	275	6.6	73	1.8	66	1.6	382	9.2	77	1.9	60	1.4	117	2.8	339	8.2	**4160**
**2011–2015**																					
Sex																					
Male	2715	63.9	182	4.3	290	6.8	67	1.6	63	1.5	373	8.8	201	4.7	80	1.9	155	3.7	121	2.9	**4247**
Female	132	30.9	35	8.2	9	2.1	6	1.4	20	4.7	86	20.1	12	2.8	9	2.1	6	1.4	112	26.2	**427**
Age (Median)	37 (IQR 28–47)		38 (IQR 29–46, p = 0.7848)		40 (IQR 30–51, p<0.001)		37 (IQR 30–50, p = 0.154)		35 (IQR 27–43, p = 0.092)		33 (IQR 28–39, p<0.001)		28 (IQR 24–34, p<0.001)		32 (IQR 28–41, p = 0.045)		32 (IQR 27–40, p<0.001)		35 (IQR 29–41, p = 0.012)		
Male to male sex	36 (IQR 28–45)		38 (IQR 29–46, p = 0.434)		39 (IQR 30–50, p<0.001)		35 (IQR 29–43, p = 0.711)		36 (IQR 30–46, p = 0.941)		32 (IQR 27–39, p<0.001)		27 (IQR 24–34, p<0.001)		31 (IQR 27–40, p = 0.204)		32 (IQR 26–39, p<0.001)		38 (IQR 30–44, p = 0.773)		
Heterosexual sex	37 (IQR 30–48)		37 (IQR 30–48, p = 0.017)		46 (IQR 32–59, p = 0.221)		50 (34–64, p = 0.084)		33 (IQR 27–41, p<0.001)		35 (IQR 30–42, p<0.001)		35 (IQR 29–39, p = 0.006)		32 (IQR 28–43, p = 0.002)		41 (29–54, p = 0.865)		35 (IQR 28–41, p<0.001)		
Exposure																					
Male to male sex	2419	66.0	149	4.1	238	6.5	53	1.5	36	1.0	335	9.1	192	5.2	52	1.4	143	3.9	50	1.4	**3667**
Heterosexual sex	428	42.5	68	6.8	61	6.1	20	2.0	47	4.7	124	12.3	21	2.1	37	3.7	18	1.8	183	18.2	**1007**
**Total**	2847	60.9	217	4.6	299	6.4	73	1.6	83	1.8	459	9.8	213	4.6	89	1.9	161	3.4	233	5.0	**4674**
**TOTAL 2006–2015**	**5440**	**61.6**	**395**	**4.5**	**574**	**6.5**	**146**	**1.7**	**149**	**1.7**	**841**	**9.5**	**290**	**3.3**	**149**	**1.7**	**278**	**3.2**	**572**	**6.5**	**8834**

Between 2006–2010 and 2011–2015, the proportion of HIV diagnoses attributed to male-to-male sex which were in men decreased from 72.5% to 66.5%, while the proportion born in South-East, North-East or Southern and Central Asia increased from 9.7% to 15.8%. The proportion attributed to heterosexual sex which were in Australian-born men and women increased from 33.7% to 42.5% while the proportion born in Sub-Saharan Africa and South-East Asia decreased from 27.2% to 18.2%, and 15.8% to 12.3% respectively.

### Age

Age at diagnosis is an important factor in understanding who is at risk and for targeting of prevention, treatment and care programs.

For diagnoses attributed to male-to-male sex, the median age at diagnosis compared to Australian-born men was significantly lower in both time periods in South-East Asian and North-East Asian born men, higher for men born in North-West Europe, and lower in 2011–2015 alone for men born in the Americas. For diagnoses attributed to heterosexual exposures, the median age at diagnosis compared to the Australian-born was significantly lower in both time periods in men and women born in Oceania, South-East Asia, Southern and Central Asia and Sub-Saharan Africa and higher in those born in North-West Europe. The median age at diagnosis compared to Australian-born was higher in 2006–2010 alone for those born in Southern and Eastern Europe and lower in 2011–2015 alone for men and women born in North Africa and the Middle East and North-East Asia. There were no significant differences in age for other region of birth or exposure groupings compared to Australian-born, in either 2006–2010 or 2011–2015.

In 2006–2010, amongst HIV diagnoses attributed to male-to-male sex, the highest proportion were among Australian-born men, followed by men born in South-East Asia, North-West Europe, Oceania, the Americas, North-East Asia, Southern and Eastern Europe, Sub-Saharan Africa, North Africa and the Middle East and Southern and Central Asia. In 2011–2015, the highest proportion were among Australian-born men, followed by men born in South-East Asia, North-West Europe, North-East Asia, Oceania, the Americas, Southern and Eastern Europe, Southern and Central Asia, Sub-Saharan Africa, and North Africa and the Middle East.

In 2006–2010, amongst HIV diagnoses attributed to heterosexual sex, the highest proportion were among Australian-born men and women, followed by those born in Sub-Saharan Africa, South-East Asia, North-West Europe, Oceania, North Africa and Middle East, Southern and Central Asia, Southern and Eastern Europe, the Americas and North-East Asia. In 2011–15, the highest proportion were among Australian-born men and women, followed by those born in Sub-Saharan Africa, South-East Asia, Oceania, North-West Europe, North Africa and Middle East, Southern and Central Asia, North-East Asia, Southern and Eastern Europe and the Americas.

### Time since arrival

There were 383 people born overseas and diagnosed with HIV in 2015. Of these, data on time since arrival was available for 197 (51.4%) diagnoses, on likely place of acquisition for 297 (77.6%) diagnoses, and on both time since arrival and likely place of acquisition for 177 (46.2%) diagnoses. The median time since arrival was three years (IQR 1–11 years) for men in whom male-to-male sex was the likely exposure, six years (IQR 1–17 years) for men and five years (IQR 1–11 years) for women in whom heterosexual sex was the likely exposure.

Of the 230 new HIV diagnoses attributed to male-to-male sex for whom data on likely place of acquisition was available, 78 (33.9%) were acquired overseas. The proportion acquired overseas was higher for HIV diagnoses attributed to heterosexual contact (47 or 70.2%, X^2^ = 28.0, p<0.001).

### Changes in HIV diagnosis rate by region of birth

Results of the Poisson regression analysis are at Table 2.

**Table 2:** HIV diagnosis rates by region of birth and exposure, with summary rate ratios, 2006–2015.

		2006–2010					2011–2015					†SRR 2006–2010 vs 2011–2015			
		Age std. rate	IRR	95% CI		p value	Age std. rate	IRR	95% CI		p value	SRR	95% CI		P value
Male-to-male sex	Australia	5.97	1				6.07	1				1.02	0.96	1.08	0.538
	Oceania	7.15	1.20	0.99	1.45	0.060	7.12	1.17	0.99	1.39	0.071	1.00	0.78	1.28	0.970
	North-West Europe	5.60	0.94	0.82	1.07	0.353	6.60	1.09	0.96	1.22	0.175	1.18	0.99	1.40	0.060
	Southern and Eastern Europe	4.05	0.68	0.55	0.83	<0.001	4.83	0.80	0.66	0.96	0.018	1.19	0.91	1.57	0.209
	North Africa and the Middle East	2.66	0.45	0.30	0.67	<0.001	3.37	0.56	0.40	0.78	0.001	1.27	0.75	2.14	0.373
	South-East Asia	11.34	1.90	1.63	2.21	<0.001	15.57	2.56	2.27	2.89	<0.001	1.37	1.14	1.65	0.001
	North-East Asia	5.16	0.86	0.68	1.11	0.249	11.23	1.85	1.59	2.15	<0.001	2.18	1.64	2.89	<0.001
	Southern and Central Asia	1.45	0.24	0.15	0.38	<0.001	2.37	0.39	0.29	0.53	<0.001	1.63	0.95	2.82	0.079
	Americas	14.17	2.37	1.91	2.95	<0.001	19.34	3.18	2.68	3.79	<0.001	1.37	1.04	1.79	0.025
	Sub-Saharan Africa	6.16	1.03	0.76	1.40	0.842	5.23	0.86	0.64	1.16	0.324	0.85	0.55	1.30	0.450
Heterosexual—Men	Australia	0.65	1				0.72	1				1.10	0.93	1.30	0.251
	Oceania	1.03	1.57	0.96	2.60	0.075	1.48	2.05	1.39	3.01	<0.001	1.43	0.78	2.64	0.247
	North-West Europe	1.08	1.65	1.20	2.26	0.002	1.32	1.84	1.39	2.42	<0.001	1.23	0.84	1.81	0.296
	Southern and Eastern Europe	0.43	0.66	0.35	1.25	0.205	0.84	1.17	0.74	1.86	0.496	1.94	0.90	4.18	0.089
	North Africa and the Middle East	2.41	3.69	2.37	5.73	<0.001	2.26	3.14	2.06	4.79	<0.001	0.94	0.52	1.69	0.832
	South-East Asia	3.39	5.19	3.86	6.96	<0.001	1.68	2.33	1.62	3.35	<0.001	0.49	0.32	0.77	0.002
	North-East Asia	0.14	0.21	0.05	0.94	0.041	0.51	0.71	0.36	1.42	0.333	3.74	0.72	19.43	0.116
	Southern and Central Asia	2.32	3.55	2.42	5.20	<0.001	1.16	1.61	1.03	2.49	0.035	0.50	0.28	0.87	0.014
	Americas	1.54	2.35	1.21	4.56	0.011	1.70	2.36	1.32	4.22	0.004	1.10	0.46	2.63	0.822
	Sub-Saharan Africa	18.85	28.82	23.31	35.64	<0.001	7.27	10.10	7.67	13.29	<0.001	0.39	0.28	0.52	0.000
Heterosexual—women	Australia	0.32	1				0.34	1				1.05	0.83	1.34	0.669
	Oceania	2.17	6.80	4.63	9.98	<0.001	1.83	5.43	3.73	7.92	<0.001	0.84	0.52	1.36	0.484
	North-West Europe	0.67	2.10	1.37	3.21	0.001	0.41	1.21	0.73	2.03	0.461	0.61	0.33	1.13	0.118
	Southern and Eastern Europe	0.39	1.22	0.60	2.48	0.585	0.71	2.10	1.22	3.61	0.008	1.81	0.77	4.29	0.177
	North Africa and the Middle East	2.65	8.28	5.16	13.29	<0.001	2.74	8.15	5.30	12.52	<0.001	1.04	0.57	1.87	0.904
	South-East Asia	4.88	15.27	11.72	19.90	<0.001	3.00	8.91	6.72	11.81	<0.001	0.61	0.45	0.83	0.002
	North-East Asia	0.38	1.19	0.51	2.77	0.693	0.53	1.57	0.83	2.95	0.165	1.39	0.50	3.90	0.531
	Southern and Central Asia	0.58	1.81	0.77	4.28	0.174	0.45	1.35	0.62	2.92	0.451	0.78	0.25	2.43	0.671
	Americas	1.34	4.18	2.04	8.57	<0.001	0.64	1.90	0.75	4.80	0.175	0.48	0.15	1.51	0.208
	Sub-Saharan Africa	20.95	65.55	51.44	83.53	<0.001	12.87	38.21	29.68	49.19	<0.001	0.61	0.48	0.79	<0.001

IRR = incidence rate ratio, SRR = summary rate ratio,

^†^2006–2010 vs. 2011–2015 SRR, based on univariate Poisson models for each region of birth with number of diagnoses as the outcome and time interval as the independent binary variables.

#### New HIV diagnoses attributed to male-to-male sex

In 2006 to 2010, age-standardised HIV diagnosis rates attributed to male-to-male sex were significantly higher in men born in South-East Asia (IRR 1.90, 95% CI 1.63–2.21) and the Americas (IRR 2.37, 95% CI 1.91–2.95) compared with Australian-born men. Rates were significantly lower compared to Australian-born men in men born in Southern and Eastern Europe (IRR 0.68, 95% CI 0.55–0.83), North Africa and the Middle East (IRR 0.45, 95% CI 0.30–0.67) and Southern and Central Asia (IRR 0.24, 95% CI 0.15–0.38), but not significantly different for men born in Oceania, North-West Europe, North-East Asia and Sub-Saharan Africa.

In 2011 to 2015, age-standardised new HIV diagnosis rates attributed to male-to-male sex were significantly higher in men born in South-East Asia (IRR 2.56, 95% CI 2.27–2.89), North-East Asia (IRR 1.85, 95% CI 1.59–2.15) and the Americas (IRR 3.18, 95% CI 2.68–3.79) compared with men born in Australia. Rates were significantly lower in men born in Southern and Eastern Europe (IRR 0.80, 95% CI 0.66–0.96), North Africa and the Middle East (IRR 0.56, 95% CI 0.40–0.78) and Southern and Central Asia (IRR 0.39, 95% CI 0.29–0.53). Rates were not significantly different for men born in Oceania, North-West Europe and Sub-Saharan Africa.

Between the time periods 2006–2010 and 2011–2015, HIV diagnosis rates attributed to male-to-male sex increased 1.37 times (95% CI 1.14–1.65) in men born in South-East Asia, 2.18 times (95% CI 1.64–2.89) in men born in North-East Asia and 1.37 times (95% CI 1.04–1.79) for men from the Americas, as expressed by the SRR. Changes for other regions of birth were not significant.

#### New HIV diagnoses in men attributed to heterosexual contact

In 2006 to 2010, age-standardised HIV diagnosis rates attributed to heterosexual contact were significantly higher in men born in North-West Europe (IRR 1.65, 95% CI 1.20–2.26), North Africa and the Middle East (IRR 3.69, 95% CI 2.37–5.73), South-East Asia (IRR 5.19, 95% CI 3.86–6.96), Southern and Central Asia (IRR 3.55, 95% CI 2.42–5.20), the Americas (IRR 2.35, 95% 1.21–4.56) and Sub-Saharan Africa (IRR 28.82, 95% 23.31–35.64) compared to Australian-born men. Rates were lower or not significantly different for men born in Southern and Eastern Europe, North-East Asia or Oceania compared with men born in Australia.

In 2011 to 2015, new HIV diagnosis rates attributed to heterosexual contact were significantly higher in men born in Oceania (IRR 2.05, 95% CI 1.39–3.01), North-West Europe (1.84, 95% CI 1.39–2.42), North Africa and the Middle East (IRR 3.14, 95% CI 2.06–4.79), South-East Asia (IRR 2.33, 95% CI 1.62–3.35), Southern and Central Asia (IRR 1.61, 95% CI 1.03–2.49), the Americas (IRR 2.36, 95% CI 1.32–4.22) and Sub-Saharan Africa (IRR 10.10, 95% CI 7.67–13.29) than in the Australian-born. Rates were not significantly different for men born in Southern and Eastern Europe or North-East Asia.

Between the time periods 2006–2010 and 2011–2015, new HIV diagnosis rates attributed to heterosexual contact decreased significantly in men born in South-East Asia (SRR 0.49, 95% CI 0.32–0.77), Southern and Central Asia (SRR 0.50, 95% CI 0.28–0.87) and Sub-Saharan Africa (SRR 0.39, 95% CI 0.28–0.52). Changes for other regions of birth were not significant.

#### New HIV diagnoses in women attributed to heterosexual contact

Age-standardised new HIV diagnosis rates attributed to heterosexual contact from 2006 to 2010 were higher than in women born in Oceania (IRR 6.80, 95% CI 4.63–9.98), North-West Europe (IRR 2.10, 95% CI 1.37–3.21) North Africa and the Middle East (IRR 8.28, 95% CI 5.16–13.29), South-East Asia (IRR 15.27, 95% CI 11.72–19.90), the Americas (IRR 4.18, 95% CI 2.04–8.57) and Sub-Saharan Africa (IRR 65.55, 95% CI 51.44–83.53) than in Australian-born women. Rates were not significantly different for women from Southern and Eastern Europe, North-East Asia and Southern and Central Asia compared with those born in Australia.

From 2011 to 2015, new HIV diagnosis rates attributed to heterosexual contact were significantly higher in women born in Oceania (IRR 5.43, 95% CI 3.73–7.92), Southern and Eastern Europe (IRR 2.10, 95% CI 1.22–3.61), North Africa and the Middle East (IRR 8.15, 95% CI 5.30–12.52), South-East Asia (IRR 8.91, 95% CI 6.72–11.81) and Sub-Saharan Africa (IRR 38.21, 95% CI 29.68–49.19) than in Australian-born women. Rates were not significantly different for other regions of birth.

Between 2006–2010 and 2011–2015, HIV diagnosis rates attributed to heterosexual contact decreased significantly in women born in South-East Asia and Sub-Saharan Africa, by 0.61 (95% CI 0.45–0.83) and 0.61 (95% CI 0.48–0.79) times respectively. Changes for other regions of birth were not significant.

### Correlation between HIV diagnoses and permanent and long-term arrivals

After adjusting for age, HIV diagnoses attributed to male-to-male sex had a significant positive association with permanent and long-term arrivals for those from North Africa and the Middle East, North-East Asia and the Americas, and a borderline significant positive association for those from Southern and Central Asia (Table 3). For male diagnoses attributed to heterosexual sex, there was a significant negative association with permanent and long-term arrivals for those from South-East Asia, and a positive association for those from Southern and Central Asia and Sub-Saharan Africa. For female diagnoses attributed to heterosexual sex, there was a significant negative association with permanent for those from South-East Asia, and a positive association for those from Oceania, Southern and Central Asia and Sub-Saharan Africa (Table 3). Associations for all other regions of birth and exposures were not significant.

**Table 3:** Univariate and multivariate (adjusting for age) regression analyses, examining the relationship between HIV diagnoses and permanent and long-term arrivals, 2006–2015.

		Univariate			Multivariate	
Exposure	Region of birth	Coefficient	R^2^	p-value	Coefficient	p-value
Male-to-male sex	Oceania	0.0004	0.11	0.35	0.0004	0.20
	North-West Europe	-0.0001	0.03	0.61	-0.0001	0.74
	Southern and Eastern Europe	-0.0002	0.04	0.57	0.0003	0.35
	North Africa and the Middle East	0.0006	0.53	0.02	0.0005	0.04
	South-East Asia	0.0008	0.06	0.50	0.0006	0.39
	North-East Asia	0.0013	0.42	0.04	0.0013	0.00
	Southern and Central Asia	0.0001	0.04	0.60	0.0001	0.06
	Americas	0.0013	0.50	0.02	0.0014	0.00
	Sub-Saharan Africa	0.0003	0.12	0.32	-0.0001	0.50
Heterosexual—men	Oceania	0.0002	0.26	0.14	0.0001	0.52
	North-West Europe	0.0001	0.07	0.45	-0.0001	0.30
	Southern and Eastern Europe	0.0001	0.03	0.65	0.0000	0.73
	North Africa and the Middle East	-0.0003	0.11	0.36	-0.0002	0.25
	South-East Asia	-0.0004	0.59	0.01	-0.0003	0.01
	North-East Asia	0.0001	0.13	0.31	0.0001	0.23
	Southern and Central Asia	0.0001	0.27	0.13	0.0001	0.02
	Americas	0.0001	0.02	0.69	0.0000	0.88
	Sub-Saharan Africa	0.0025	0.76	0.00	0.0022	0.00
Heterosexual—women	Oceania	0.0004	0.25	0.14	0.0004	0.03
	North-West Europe	-0.0001	0.15	0.27	-0.0001	0.14
	Southern and Eastern Europe	0.0000	0.00	0.85	0.0000	0.91
	North Africa and the Middle East	-0.0003	0.14	0.28	-0.0003	0.15
	South-East Asia	-0.0005	0.47	0.03	-0.0007	0.00
	North-East Asia	0.0000	0.00	0.90	0.0000	0.64
	Southern and Central Asia	0.0000	0.17	0.23	0.0001	0.04
	Americas	0.0001	0.12	0.33	0.0001	0.49
	Sub-Saharan Africa	0.0011	0.21	0.18	0.0007	0.29

## Discussion

The epidemiology of HIV in Australia is changing. While HIV diagnosis rates for Australian-born men and women in all exposure categories have been stable, rates attributed to male-to-male sexual exposure among men born in South-East Asia, North-East Asia and the Americas increased significantly between 2006–2010 and 2011–2015, and are higher than those of Australian-born men. In 2011–2015 Asian-born men (from South-East Asia, North-East and Southern and Central Asia) accounted for over 15% of all new HIV diagnoses attributed to male-to-male sex in Australia. Diagnosis rates attributed to heterosexual exposure amongst men and women born in South-East Asia and Sub-Saharan Africa, and men born in Southern and Central Asia, decreased over the ten years but remained higher than rates in Australian-born people.

Migration patterns and the epidemiology of HIV in countries of origin may partially explain these changes. With respect to male-to-male sexual exposure, increases in the number of new arrivals were significantly correlated with increases in new HIV diagnoses for those from North Africa and the Middle East, North-East Asia, Southern and Central Asia and the Americas. In relation to heterosexual exposure, increases in new arrivals were associated with increases in new HIV diagnoses in men from Sub-Saharan Africa, and decreases in new HIV diagnoses for men and women from South-East Asia.

These trends are consistent with the presence of concentrated epidemics among men who have sex with men (MSM) in countries in North-East Asia, Southern and Central Asia and the Americas; and with changes in South-East Asian countries such as Thailand and Cambodia from generalised to concentrated epidemics amongst populations such as MSM [16]. Findings are also consistent with other high-income countries in Europe, where decreases in heterosexually acquired HIV amongst Sub-Saharan Africa born men and women and increases in new HIV diagnoses among MSM from other regions such as Central Asia, corresponding with migration patterns, have also been observed [1, 2]. Studies of other infectious diseases such as tuberculosis and hepatitis B have consistently found an association between migration patterns and prevalence in high income countries [17, 18]. However for HIV findings are mixed, with a recent study comparing migration intensity and peak national HIV prevalence in 141 countries finding no association, and highlighting the limitation of ecological analyses which do not distinguish between infection acquired prior to or after arrival in host country [19].

A positive association between new arrivals and new HIV diagnoses does not mean people born overseas acquired HIV prior to migration. Data for 2015 suggest overseas-born men diagnosed with HIV attributed to male-to-male sex are more likely overseas-born men and women diagnosed with HIV attributed to heterosexual sex to have acquired the infection in Australia. Recent European studies also show overseas-born MSM diagnosed with HIV are now more likely to have acquired the infection post migration [20, 21]. More research is needed to understand the HIV-related risks and vulnerabilities of overseas-born MSM, including potential lower levels of health literacy, and awareness of prevention strategies available in Australia and how to access them. Behavioural and programmatic data suggest overseas-born MSM are less likely to access pre-exposure prophylaxis (PrEP), compared with Australian-born MSM [22] but are as likely to engage in behaviours such as condom-less anal sex [23].

Asian-born men diagnosed with HIV may have recently arrived in Australia [24], potentially without permanent residency status or access to publicly funded treatment. Students coming from countries such as China and Thailand also continue to increase [25], suggesting this may be an important sub-group at risk of HIV. Facilitating timely testing and treatment initiation through various modalities, including sexual health services and educational settings, is important. Promoting access to, and uptake of, new prevention modalities such as pre-exposure prophylaxis can also help reduce new diagnoses. Recent efforts to increase enrolment among Asian-born MSM in PrEP trials have also highlighted the importance of ensuring health promotion is conducted in community languages [M Vaughan, personal communication 20 August 2018].

The strength of this study is the use of population-wide data on new HIV diagnoses and new arrivals, and high level of completeness of key variables such as country of birth in the National HIV Registry. Limitations include first, the use of the overall male population as the denominator in analyses for diagnoses in men attributed to male-to-male sex, as there are no population estimates for MSM disaggregated by region of birth. HIV diagnosis rates attributed to male-to-male sex across all regions of birth have therefore been underestimated. The analysis of association with migration patterns also assumes MSM from different regions of birth are as likely to migrate as are heterosexual men.

Secondly, the National HIV registry does not collect information on visa status, so we could not disaggregate cases by length of stay or visa category. Data on place of acquisition and year of arrival was only available for 2015, and incomplete. Data from future years on these variables is needed to verify the findings. Finally, we purposely chose to present data by region rather than country of birth to avoid stigmatising particular communities, however this analysis may mask differences within a given region. Numbers for some regions such as the Southern and Central Asia remain small, so findings should be interpreted with caution.

Further research is needed into HIV risk amongst Asian-born MSM, their social and sexual networks, behavioural and cultural factors which might increase risk amongst different sub-groups and communities, and factors influencing the uptake of HIV prevention, testing and treatment measures, to better tailor interventions for this group. Existing evidence suggests integration of provider-initiated testing and counselling into primary care is a promising approach [26], as is ensuring interventions are holistic, and account for the economic, cultural and psycho-social context for ethnic minority MSM [27].

## Conclusion

The epidemiology of HIV amongst overseas-born populations in Australia is complex and changing. HIV programs must adapt to emerging groups at risk, such as Asian-born MSM, while still effectively targeting groups such as South-East Asian and Sub-Saharan African born heterosexual men and women who continue to experience high HIV diagnosis rates.
